# The Plant Circadian Clock and Chromatin Modifications

**DOI:** 10.3390/genes9110561

**Published:** 2018-11-20

**Authors:** Ping Yang, Jianhao Wang, Fu-Yu Huang, Songguang Yang, Keqiang Wu

**Affiliations:** 1 Key Laboratory of South China Agricultural Plant Molecular Analysis and Genetic Improvement, South China Botanical Garden, Chinese Academy of Sciences, Guangzhou 510650, China; yefeng77@sohu.com (P.Y.); wangjianhao@scbg.ac.cn (J.W.); 2 University of Chinese Academy of Sciences, Chinese Academy of Sciences, Beijing 100049, China; 3 Institute of Plant Biology, National Taiwan University, Taipei 106, Taiwan; clautus@mac.com

**Keywords:** circadian clock, oscillators, transcriptional and post-transcriptional regulation, chromatin modifications, *Arabidopsis*

## Abstract

The circadian clock is an endogenous timekeeping network that integrates environmental signals with internal cues to coordinate diverse physiological processes. The circadian function depends on the precise regulation of rhythmic gene expression at the core of the oscillators. In addition to the well-characterized transcriptional feedback regulation of several clock components, additional regulatory mechanisms, such as alternative splicing, regulation of protein stability, and chromatin modifications are beginning to emerge. In this review, we discuss recent findings in the regulation of the circadian clock function in *Arabidopsis thaliana*. The involvement of chromatin modifications in the regulation of the core circadian clock genes is also discussed.

## 1. Introduction

**Figure 1:**
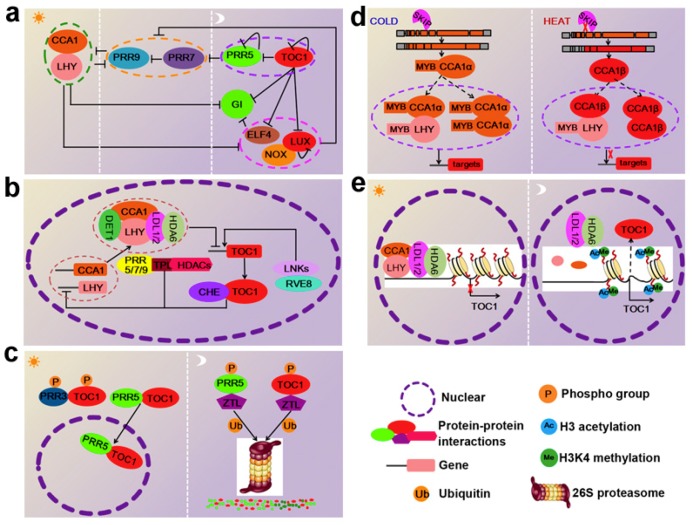
Multiple layers of regulation refine circadian clock activity in *Arabidopsis thaliana*. (**a**). Transcriptional feedback loops at the core of the circadian oscillator in *Arabidopsis*: At dawn, the expression of the pseudo-response regulator (PRR)-encoding genes; GIGANTEA (GI); timing of CAB expression 1 (TOC1); and the evening complex EC members LUX ARRHYTHMO (LUX), NOX, and early flowering 4 (ELF4) were repressed by circadian clock associated 1 (CCA1) and late elongated hypocotyl (LHY). PRR9, PRR7, PRR5, and TOC1 are sequentially expressed and repress the expression of CCA1 and LHY, as well as their own transcription. In the evening, all of the previously expressed components are repressed by TOC1. Subsequently, the EC maintains the repression of GI, PRR9 and PRR7. (**b**) Protein–protein interactions among clock components: The homo- or heterodimerization of CCA1 and LHY represses evening-phased genes by binding to an evening element in their promoters. To repress gene targets, circadian clock associated 1 (CCA1) and late elongated hypocotyl (LHY) require de-etiolated 1 (DET1), Histone deacetylase 6 (HDA6), and lysine-specific demethylase 1-like 1 and 2 (LDL1/2) as corepressors. Sequentially, the PRRs (PRR9, PRR7, and PRR5) bind to the CCA1 and LHY promoters and recruit TOPOLESS TPL and HDA6, thereby inhibiting the transcription of CCA1 and LHY. The interaction between TOC1 and CCA1 hiking expedition (CHE) helps TOC1 bind to the promoters of CCA1 and LHY. Additionally, night light-inducible and clock-regulated gene 1s (LNKs) interact with CCA1-like 5 (RVE8) and act as coactivators inducing the expression of TOC1. (**c**) Protein stability and turnover modulate the activity of oscillators: in the afternoon, the ZEITLUPE (ZTL)-mediated proteasomal degradation of TOC1 is interrupted by ZTL’s interaction with PRR3 (which hinders ZTL access) and PRR5 (which promotes TOC1 translocation to the nucleus). On the other hand, the phosphorylation of PRR5 and TOC1 enhances their binding to ZTL, which promotes their degradation later in the evening. (**d**) The alternative splicing regulates activity of CCA1: under high temperatures, the SNW/ski-interacting protein (SKIP) mediates CCA1 alternative splicing, leading to an aberrant spliced form, CCA1β, due to the fourth intron retention. CCA1β encodes a shorter protein lacking the DNA-binding Myb domain that is still able to homo/heterodimerize with the functional CCA1α and LHY. However, CCA1β/CCA1α and CCA1β/LHY dimers show reduced DNA binding activity to downstream targets. Under cold conditions, the accumulation of the correctly spliced variant CCA1α leads to increased CCA1 protein levels. Fully functional CCA1/CCA1 and CCA1/LHY dimers are thus able to bind to the promoters of targets. (**e**) Regulation of clock central oscillator TOC1 by chromatin modifications: in the morning, accumulated-CCA1/LHY, associated with the histone modification complex containing LDL1/2 and HDA6, attaches to the promoter of TOC1, thereby reducing the H3Ac and H3K4Me levels of TOC1. Consequently, TOC1 expression is low. In the evening, CCA1 and LHY expression are low, while TOC1 is highly expressed because the LDL1/2-HDA6 complex is released from the TOC1 promoter.

Plants, like animals, exhibit rhythmic biological activity, with a periodicity of 24 h. The rhythms are known as the circadian clock, which allows plants to coordinate the temporal organization of biological processes to specific times of the day or night [1]. The core components of the circadian system are oscillators, which are responsible for generating circadian rhythms. In addition to the central oscillators, the circadian system involves the input pathways that integrate oscillators’ functions with environmental timing stimuli, such as light and temperature, and the output pathways that provide a link between the oscillators and the diverse biological processes [2]. In the model plant *Arabidopsis thaliana*, more than 20 clock or clock-associated components are composed of interlocking transcription-translation feedback loops, which appears to be significantly more complicated than that of other model eukaryotes, such as *Drosophila* [3]. Different components of the circadian system act at distinct times throughout the day and night to reciprocally regulate expression of other clock genes at both the transcriptional and post-transcriptional levels (Figure 1):

The orange star and light arch represent day and night, respectively. Ovals represent functional groups. Black bars indicate repression, and black arrows indicate the activation of transcription. Broken lines indicate relationships not proven to be direct or detected only under specific conditions

## 2. Transcriptional Regulation of the Circadian Clock Genes

In plants, the circadian oscillator comprises the morning and evening loops that have been proposed to form a negative feedback loop to generate circadian rhythms [4]. In the morning loop, two key single-MYB-domain-containing transcription factors, CIRCADIAN CLOCK ASSOCIATED 1 (CCA1) [5] and LATE ELONGATED HYPOCOTYL (LHY) [6], together with pseudo-response regulators 5, 7, and 9 (PRR5, PRR7, and PRR9) [7] interlock, and the core evening loop is composed of TIMING OF CAB EXPRESSION 1 (TOC1, also known as PRR1) [7,8]. Indeed, as two physically interacting and partially redundant proteins, the transcription and protein levels of CCA1 and LHY are highest in the morning [9,10]. CCA1 and LHY form a heterodimer in order to repress the expression of evening genes [11,12,13]. In addition, they also repress each other’s expression in an accurate timekeeping manner [5,6], since the single loss of function of either CCA1 or LHY causes a shortening of the period under free-running conditions, while the *cca1 lhy* double mutant is arrhythmic [14]. The first evening clock gene known to be repressed by CCA1 and LHY is *TIMING OF CAB EXPRESSION 1* (*TOC1*, also known as *PRR1*), a member of the *PRR* family whose mutation shortens the period [8] by binding to a motif within the *TOC1* promoter called the evening element (EE) [15]. Interestingly, the EE motif is always found in clock-regulated gene promoters [12], and also mediates cold responses [16]. The different signals converged at the EE possibly involve the binding of alternative transcription factor complexes, since CCA1 and LHY can form heterodimers [9,10], as well as the presence of protein–EE complexes, even in the absence of CCA1 and LHY [2]. In addition to *TOC1*, several other members of the *PRR* family, including *PRR7* and *PRR9*, are also repressed by CCA1 and LHY [13,17]. Furthermore, several other evening genes, including *GIGANTEA* (*GI*), *LUX ARRHYTHMO* (*LUX*, an MYB-like transcription factor), and *BROTHER OF LUX ARRHYTHMO* (*BOA*, also known as *NOX*), as well as *EARLY FLOWERING 3* (*ELF3*) and *ELF4* (two unrelated novel nuclear proteins) are also repressed by CCA1 and LHY [18,19,20].

The expression of *CCA1* and *LHY* is also repressed by *TOC1*, *PRR7*, and *PRR9* [21,22,23]. After dawn, *PRR9* is the first to be expressed, followed consecutively by *PRR7* and *PRR5* in the afternoon and finally by *TOC1* in the evening [24]. Thus, this mechanism ensures that *CCA1* and *LHY* are only expressed during a narrow window of time near dawn [25]. The repression of *CCA1* caused by *TOC1* involves the interaction between TOC1 and CCA1 HIKING EXPEDITION (CHE) in an as-yet undefined manner [26]. Furthermore, *ELF4*, *ELF3*, and *LUX* form an “evening complex” to repress the expression of the day-phased clock gene *PRR9* [27,28,29,30]; mutation of any member of this complex causes plants to become arrhythmic [20]. Several genes homologous to *CCA1* and *LHY*, such as *REVEILLE8* (*RVE8*, also known as *LHY* and *CCA1*-*like 5*, or *LCL5*) are also repressed by *PRR*s [31,32]. However, unlike the repressors CCA1 and LHY, RVE8 promotes hundreds of evening genes that contain EEs in their promoters, such as *PRR5*, *TOC1*, *GI*, *ELF4*, and *LUX* [32,33]. Furthermore, *RVE4* and *RVE6*, two close homologs of *RVE8*, also play partially redundant roles in the circadian system, since the *rve4 rve6 rve8* triple mutant not only exhibits a more extreme long-period phenotype than the *rve8* single mutant, but also loses the predominantly afternoon-phased EE binding activity [33].

## 3. Post-Transcriptional Regulation of the Circadian Clock Genes

In addition to transcriptional regulation, many post-transcriptional regulatory mechanisms, such as alternative splicing (AS) and regulation of protein stability, are key to plant circadian oscillators. Many clock genes, including *CCA1*, *LHY*, *RVE8*, *TOC1*, *ELF3*, and *GI* are regulated by alternative splicing [34,35,36]. The abundance of the different splice variants is regulated by temperature [34,37], photoperiod, salt stress, and light [36,38], supporting the idea that AS is an important mechanistic link between environmental signals and clock performance. Indeed, the splice variant of *CCA1* with a retained fourth intron produces a protein called CCA1β, which lacks the MYB-like DNA-binding domain and is suppressed under cold conditions [37]. CCA1β can interrupt the function of full-length CCA1 (CCA1α) by competing with CCA1α and LHY in the formation of nonfunctional homo- and heterodimers [37]. In turn, an *LHY* splice variant with a premature stop codon accumulates at low temperatures [34], indicating a role for AS in maintaining an appropriate balance between *CCA1* and *LHY* during cold acclimation. Moreover, spliceosome regulators that affect plant clock function have been reported. For example, protein arginine methyltransferease 5 (PRMT5), a conserved methyltransferase involved in the methylation of histones, regulates the alternative splicing of *PRR9* [39,40], presumably via PRMT5-dependent methylation of splicing factors [41]. Another protein involved in mRNA splicing is SNW/Ski-interacting protein (SKIP), a component of the spliceosome. The SKIP also regulates the AS of *PRR9* and other clock genes including *PRR7*, *CCA1*, *LHY*, and *TOC1* [35], and loss of *SKIP* causes a long period phenotype [35]. Additionally, the mutation of *SPLICEOSOMAL TIMEKEEPER LOCUS* 1 (*STIPL1*), a homolog of a human spliceosomal protein involved in spliceosome disassembly, causes a long-period phenotype [42]. In *stipl1* mutants, the accumulation of splicing variants of *CCA1*, *LHY*, *TOC1*, and *PRR9* transcript is altered [42].

Protein–protein interaction networks are also critical for the regulation of circadian clock functions. Indeed, the repressive activity of CCA1 and LHY on evening genes depends on DE-ETIOLATED1 (DET1), a repressor of photomorphogenesis [43]. Similarly, PRR9, PRR7, and PRR5 physically interact with a transcriptional co-repressor, TOPLESS, and this interaction is required for the transcriptional repression of *CCA1* and *LHY* [44]. The activity of this repressed complex seems to be required for TPL-dependent recruitment of histone deacetylase 6 (HDA6) [44]. A recent report demonstrated that lysine-specific demethylase 1 (LSD1)-like histone demethylases LDL1 and LDL2 form a histone modification complex with the histone deacetylase HDA6. The LDL1/LDL2–HDA6 histone modification complex is functionally associated with CCA1/LHY in the regulation of circadian clock genes [45]. Additionally, two clock-regulated and light-induced proteins, night light-inducible and clock-regulated 1 and 2 proteins (LNK1 and LNK2), can interact with CCA1, LHY, RVE4, and RVE8 [46,47]. In fact, the recruitment of LNK1 to the *PRR5* and *TOC1* promoters happens via interaction with RVE4 and RVE8, and the activation of *PRR5* as well as *TOC1* transcription by RVE8 requires LNK1 and LNK2 as transcriptional coactivators [46].

As a common mechanism for modulating protein stability, ubiquitination is also involved in the degradation of clock proteins. The F-box protein ZEITLUPE (ZTL) and its homologs—flavin binding, Kelch repeat, F-box (FKF1), and LOV KELCH protein 2 (LKP2)—have been characterized as circadian clock regulators in *Arabidopsis*. ZTL, with a blue light photosensor LOV (light, oxygen, and voltage) domain and a KELCH protein–protein interaction domain, targets TOC1 and PRR5 for ubiquitination and subsequent proteasome degradation [48,49]. Similarly, FKF1 and LKP2 also contribute to shaping PRR5 and TOC1 protein oscillations through direct interaction and degradation [48,50]. GI physically interacts with ZTL and stabilizes it, while ZTL reciprocally controls GI stability and nucleocytoplasmic partitioning [51]. Finally, the degradation of GI is promoted via ELF3-mediated interaction with the E3-ubiquitin ligase constitutive photomorphogenic 1 (COP1), thereby also triggering the ubiquitination and degradation of the substrate adaptor ELF3 [52].

Phosphorylation has also been demonstrated to affect clock protein stability and interactions with other proteins. Indeed, elevated phosphorylation of all PRR proteins, leading to the degradation of many PRRs, has been observed [53]. Phosphorylation of PRR5 and TOC1 enhances each protein’s interactions with ZTL, thereby promoting each one’s subsequent degradation [53]. In contrast, PRR5 interacting with TOC1 enhances PRR5’s accumulation in the nucleus, and prevents it from being targeted for degradation by ZTL, which is exclusively found in the cytoplasm [50].

## 4. Involvement of the Circadian Clock Regulation by Chromatin Modifications

In eukaryotic cells, genomic DNA is packaged with histones to form a complex structure known as chromatin. Thus, the chromatin structure is directly linked to the regulation of gene expression in response to developmental and environmental cues, through the modulation and accessibility of transcriptional regulatory proteins [54]. Multiple chemical and reversible modifications of DNA and histones, such as DNA methylation, histone acetylation, and histone methylation regulate chromatin activity and function [54,55]. To date, accumulating evidence reveals that changes in chromatin structure modulate circadian function (Table 1).

For example, the expression of *CCA1* correlates with methylation levels of CHH (where H = A, T, or C) sites in the promoter region in heterosis [56]. In contrast, the expression of *TOC1* is accompanied by the clock-controlled pattern of histone acetylation [57]. Indeed, at dawn, *TOC1* expression is repressed by *CCA1* via the binding of CCA1 to the *TOC1* promoter, which depends on a repressive chromatin environment promoted by histone H3 deacetylation [57]. Recent studies indicated that HDA6 is involved in this process [45]. Unlike CCA1, which favors histone hypoacetylation, RVE8/LCL5 leads to H3 hyperacetylation at the *TOC1* promoter, since overexpression of *RVE8*/*LCL5* results in a rising phase of *TOC1* expression, which in turn coincides with increased H3 acetylation [58].

In addition to *TOC1*, the regulation of other oscillators by oscillating histone marks is also investigated. For instance, histone acetylation (H3ac, H3K9/H3K27ac, H3K56ac, and H3K9/ 14ac) is closely correlated with the rhythmic expression of *LHY*, *CCA1*, and *TOC1* [57,59,60,61], as well as *PRR5*, *PRR9*, *PRR7*, *GI*, and *LUX* [59,62]. Recent studies showed that the H3ac level of *PRR5* and *LUX* loci is regulated by HAF2, a histone acetyltransferase belonging to the TAFII250 family [62]. Interestingly, a correlation of increased H3K9ac and H4ac levels with the up-regulation of *LHY* was observed in *hda19* mutants [63]. Furthermore, the histone deacetylase HDA6 represses the expression of *TOC1* by histone deacetylation [45]. These studies indicate that both HDA6 and HDA19 are involved in the regulation of circadian functions.

The molecular components responsible for the histone methylation at the core of the plant clock are just beginning to emerge. The H3K4me3 accumulation at the promoters of *LHY*, *CCA1*, and *TOC1* is positively correlated with the rhythmic transcript levels of these genes, regulated by the histone methyltransferase SDG2/ATXR3 (set domain group 2/*Arabidopsis* trithorax-related) [60,64,65,66]. In contrast, the histone H3K36me2 levels and transcription levels of these genes show a negative correlation [61]. More recently, it was found that CCA1 and LHY recruit the histone modification complex containing LDL1/2 and HDA6 to the *TOC1* locus to reduce H3ac and H3K4Me2 levels, resulting the repression of *TOC1* [45]. The Jumonji C domain–containing histone demethylase, JMJ30/JMJD5, is also involved in circadian control. As a demethylase of H3K9me3 [67], JMJ30/JMJD5 is directly repressed by the core oscillators *CCA1* and *LHY* [68]. In turn, JMJ30/JMJD5 also promotes expression of *CCA1* and *LHY*, presumably through modulating the H3K9me3 levels at the promoter of these genes [67,68]. Interestingly, the *Arabidopsis* JMJD5/JMJ30 can rescue the circadian phenotypes of the mammalian cells that are deficient for the human JMJD5/JMJ30, suggesting a conserved function of JMJ30/JMJD5 in plants and animals [69].

Histone monoubiquitination and phosphorylation were also found to be involved in regulating circadian function. Histone monoubiquitination 1 (HUB1), an E3 ligase, controls H2B monoubiquitination (H2BUb) [70]. H2BUb is associated with H3K4me3 accumulation at several clock-controlling genes and alters their amplitude [71,72], indicating that this chromatin regulator participates in circadian rhythmicity. However, the H2BUb levels of oscillator genes have not been investigated. Moreover, chromatin immunoprecipitation-sequencing data shows that core oscillators like *CCA1* and *LHY* have increased levels of histone H3 phosphorylation on serine 28 (H3S28ph) at the end of the night, while *TOC1*, *PRR5*, and *GI* have high levels of H3S28ph at the end of the day [59]. A recent report demonstrated that MUT9P-like-kinase (MLK4), which phosphorylates histone H2A on serine 95 (H2AS95ph), interacts with CCA1, allowing MLK4 to bind to the *GI* promoter [73]. Meanwhile, CCA1 also interacts with YAF9a, a co-subunit of the Swi2/Snf2-related ATPase (SWR1) and NuA4 complexes, which themselves are responsible for incorporating the histone variant H2A.Z into chromatin and possess histone H4 acetylase activity [74,75]. Thus, decreased H2AS95ph, along with the attenuated accumulation of H2A.Z and the acetylation of H4, leads to reduced *GI* expression [73].

Interestingly, a previous study showed that *TOC1* circadian induction is accompanied by clock-controlled cycles of histone acetylation that favor transcriptionally permissive chromatin structures at the *TOC1* locus [60]. Further data shows that H3 activating marks, such as H3K9/K14Ac and H3K4Me3, associating with the transcriptional start sites (TSSs) of *CCA1*/*LHY* and *TOC1* are also circadian regulated [59]. These results seem to indicate that circadian-regulated histone activating marks lead to the circadian expression profiles of oscillators. In agreement with a recent study, the expression of some chromatin remodeling factor genes, such as *HAG3*, *HDA2/6*, *SUVH3*, and *JMJ28* displayed circadian oscillation [76]. However, in the CCA1-overexpression plants, the H3K9/14Ac and H3K4Me3 levels associated with *CCA1*, *LHY*, and *TOC1* TSSs were reduced [59], suggesting that the circadian oscillators may also affect histone modifications. Our recent results showed that CCA1/LHY recruits the LDL1/LDL2-HDA6 histone modification complex, reducing the H3Ac and H3K4Me levels in the promoter of *TOC1* [45] and indicating that circadian oscillators are key factors in histone modifications regulation. Nevertheless, considering the complexity of the circadian regulatory network, further studies are required to deepen our mechanistic understanding of circadian regulation and histone modification.

## 5. Concluding Remarks

As an integral part of plant biology, the circadian system coordinates external stimuli and an internal timing mechanism, in order to optimize growth and development. However, despite emerging evidence on new components involved in alternative splicing, regulation of protein stability, and histone modifications responsible for regulation of the core clock components, many questions still persist over the mechanism of circadian regulation. The repressive mechanism of the core components CCA1/LHY and TOC1 is still poorly understood. CCA1/LHY have been recently shown to recruit a histone modification complex containing LDL1/2 and HDA6 to their target loci (such as *TOC1*) to repress gene expression by histone deacetylation and H3K4 demethylation [45]. These findings indicate a link between chromatin modifications and the repressive mechanisms of a plant circadian rhythm’s core components. Nevertheless, transcriptional regulation associated with chromatin modifications is only one level nestled within a multi-layered regulatory network. Post-transcriptional mechanisms, protein–protein interactions, and protein degradation all have their own essential roles in aiding rhythm generation [76,77,78,79]. It is only through the integration of all of these layers of activity that plants generate a comprehensive circadian network sustaining robust rhythms. Thus, further investigation into the cross-talk between multi-layered regulatory networks will help us to understand how plants thrive in an unpredictable and changing environment.

## Figures and Tables

**Table 1:** Regulation of the core circadian clock genes by chromatin modifications in *Arabidopsis*.

Process	Histone Mark	Chromatin Modifier	Core Clock Component	References
DNA Methylation		Unknown	*CCA1*	[56]
Histone Acetylation	H3K9/H3K27ac	Unknown	*CCA1*, *LHY*, *TOC1*, *PRR5*, *GI*	[59]
	H3K56ac	Unknown	*LHY*, *PRR9*, *CCA1*,	[66]
			*PRR7*, *TOC1*, *LUX*	
	H3K9/14ac	Unknown	*CCA1*, *LHY*, *TOC1*	[60]
	H3ac	Unknown	*CCA1*, *LHY*, *TOC1*	[57,61]
	H3ac	HAF2	*PRR5*, *LUX*	[62]
Histone Deacetylation	H3K9ac/H4ac	HDA19	*LHY*	[63]
	H3ac	HDA6	*TOC1*	[45]
Histone Methylation	H3K4Me3	SDG2/ATXR3	*LHY*, *PRR9*, *CCA1*,	[60,61,66]
			*PRR7*, *TOC1*, *LUX*	
	H3K36me2	Unknown	*LHY*, *CCA1*, *TOC1*	[61]
Histone Demethylation	H3K4Me2	LDL1, LDL2	*TOC1*	[45]
	H3K9Me3	JMJ30/JMJD5	*CCA1*, *LHY*	[67,68,69]
Histone Monoubiquitination	H2BUb	HUB1		[71,72]
Histone Phosphorylation	H3S28ph	Unknown	*CCA1*, *LHY*, *TOC1*, *PRR5*, *GI*	[59]
	H2AS95ph	MLK4	*GI*	[73]

## Abbreviations

AS	Alternative splicing	BOA	Brother of LUX ARRHYTHMO
CCA1	Circadian clock associated 1	CHE	CCA1 hiking expedition
COP1	E3-ubiquitin ligase, Constitutive photomorphogenic 1	DET1	De-etiolated 1
EE	Evening element	ELF3	Early flowering 3
FKF1	Flavin binding, KELCH repeat, F-BOX	GI	GIGANTEA
HDA6	Histone deacetylase 6	HUB1	Histone monoubiquitination 1
HAG3	Histone acetyltransferase 8	HDA3	Histone deacetylase 3
SUVH3	SU(VAR)3-9 HOMOLOG 3	JMJ28	Jumonji C domain–containing histone demethylase 28
JMJ3	Jumonji C domain–containing histone demethylase 3	LHY	Late elongated hypocotyl
LDL1	LSD1-Like 1	LDL2	LSD1-Like 2
LUX	LUX ARRHYTHMO	LSD1	Lysine-Specific Demethylase 1
LNK	Night light-inducible and clock-regulated gene 1	LKP2	LOV KELCH protein 2
LOV	Light, Oxygen, and Voltage	MLK4	MUT9P-like-kinase
PRR5	Pseudo-response regulators 5	PRMT5	Protein arginine methyltransferase 5
RVE8/LCL5	CCA1-like 5	SKIP	SNW/Ski-interacting protein
STIPL1	Spliceosomal timekeeper locus 1	SDG2/ATXR3	Set domain group 1/*Arabidopsis* trithorax-related
SWR1	Swi2/Snf2-related ATPase	TOC1 (PRR1)	Timing of CAB expression 1
YAF9	a co-subunit of the SWR1	ZTL	ZEITLUPE
